# The histological and molecular characteristics of early-onset colorectal cancer: a systematic review and meta-analysis

**DOI:** 10.3389/fonc.2024.1349572

**Published:** 2024-04-26

**Authors:** Thomas Lawler, Lisa Parlato, Shaneda Warren Andersen

**Affiliations:** ^1^ School of Medicine and Public Health, Carbone Cancer Center, University of Wisconsin-Madison, Madison, WI, United States; ^2^ School of Medicine and Public Health, Department of Population Health Sciences, University of Wisconsin-Madison, Madison, WI, United States

**Keywords:** colorectal cancer, colon cancer, rectal cancer, early-onset, oncogenes, prognosis, molecular characteristics

## Abstract

**Background:**

Early-onset colorectal cancer (CRC), defined as diagnosis before age 50, has increased in recent decades. Although more often diagnosed at advanced stage, associations with other histological and molecular markers that impact prognosis and treatment remain to be clarified. We conducted a systematic review and meta-analysis concerning the prevalence of prognostic and predictive tumor markers for early- vs. late-onset CRC, including oncogene mutations, microsatellite instability (MSI), and emerging markers including immune cells and the consensus molecular subtypes.

**Methods:**

We systematically searched PubMed for original research articles published between April 2013–January 2024. Included studies compared the prevalence of tumor markers in early- vs. late-onset CRC. A meta-analysis was completed and summary odds ratios (ORs) with 95% confidence intervals (CIs) were obtained from a random effects model via inverse variance weighting. A sensitivity analysis was completed to restrict the meta-analysis to studies that excluded individuals with Lynch syndrome, a hereditary condition that influences the distribution of tumor markers for early-onset CRC.

**Results:**

In total, 149 articles were identified. Tumors from early-onset CRC are less likely to include mutations in *KRAS* (OR, 95% CI: 0.91, 0.85-0.98), *BRAF* (0.63, 0.51-0.78), *APC* (0.70, 0.58-0.84), and *NRAS* (0.88, 0.78-1.00) but more likely to include mutations in *PTEN* (1.68, 1.04-2.73) and *TP53* (1.34, 1.24-1.45). After limiting to studies that excluded Lynch syndrome, the associations between early-onset CRC and *BRAF* (0.77, 0.64-0.92) and *APC* mutation (0.81, 0.67-0.97) were attenuated, while an inverse association with *PIK3CA* mutation was also observed (0.88, 0.78-0.99). Early-onset tumors are less likely to develop along the CpG Island Methylator Phenotype pathway (0.24, 0.10-0.57), but more likely to possess adverse histological features including high tumor grade (1.20, 1.15-1.25), and mucinous (1.22, 1.16-1.27) or signet ring histology (2.32, 2.08-2.57). A positive association with MSI status (1.31, 1.11-1.56) was also identified. Associations with immune markers and the consensus molecular subtypes are inconsistent.

**Discussion:**

A lower prevalence of mutations in *KRAS* and *BRAF* is consistent with extended survival and superior response to targeted therapies for metastatic disease. Conversely, early-onset CRC is associated with aggressive histological subtypes and *TP53* and *PTEN* mutations, which may serve as therapeutic targets.

## 1 Introduction

Colorectal cancer (CRC) is the second leading cause of cancer mortality in the United States (1). The incidence of CRC has steadily declined since the 1980s, largely attributed to greater uptake of colonoscopy screening by adults aged 50 years and older (2). Concurrently, the incidence of sporadic early-onset CRC, generally defined as CRC diagnosis before age 50 without an underlying hereditary cause, has significantly increased since the mid-1990s (2). Data from the Surveillance, Epidemiology, and End Results (SEER) program reflect a 2-3% annual increase in the incidence of early-onset CRC (3). The elevated incidence of early-onset CRC may be explained by birth cohort effects where more recent birth cohorts have increased prevalence of obesity and type 2 diabetes, lower levels of physical activity, and more often consume western-style diets characterized by lower consumption of fruits and vegetables (4), as well as changes in the composition of the gut microbiome (2). While early-onset CRC may be caused by hereditary conditions defined by germline mutations in DNA mismatch-repair genes (i.e. Lynch syndrome) or in the tumor suppressor *APC* (i.e. familial adenomatous polyposis) (5), these inherited conditions account for a relatively small percentage of early-onset CRC and do not explain the increased prevalence observed in recent decades (2).

CRC is a heterogeneous disease and the clinicopathological and molecular characteristics of tumors may influence prognosis and response to treatment (6). Beyond tumor stage, multiple potential prognostic and predictive markers have been identified, including mutations in oncogenes such as *KRAS*, *BRAF*, *PIK3CA*, and *TP53*, histological subtypes including mucinous and signet ring carcinomas, and the microsatellite instability (MSI) phenotype (7). Further, several novel prognostic markers have recently been identified, including immune markers in the tumor microenvironment (8) and the CRC consensus molecular subtypes (9). It is anticipated that the continued characterization of molecular phenotypes in CRC will augment traditional clinical markers for therapeutic decision making and support the development of targeted approaches to treatment (10).

Given the increasing rate of early-onset CRC, recent publications have highlighted potential differences in the clinicopathological and molecular characteristics of tumors based on age of onset (11–14). However, it is currently unclear whether early-onset CRC is distinct from late-onset disease in terms of molecular characteristics and tumor developmental pathways (15). Understanding the molecular characteristics of early-onset CRC is necessary to guide the development of therapeutic approaches for this condition and to address underlying causes. Therefore, we have completed a systematic review and meta-analysis to comprehensively summarize the evidence linking early-onset CRC to differences in prognostic and predictive tumor markers, including oncogene mutations, histological subtypes, MSI status, as well as anti-tumor immunity and the consensus molecular subtypes.

## 2 Methods

### 2.1 Literature review

**Figure 1:**
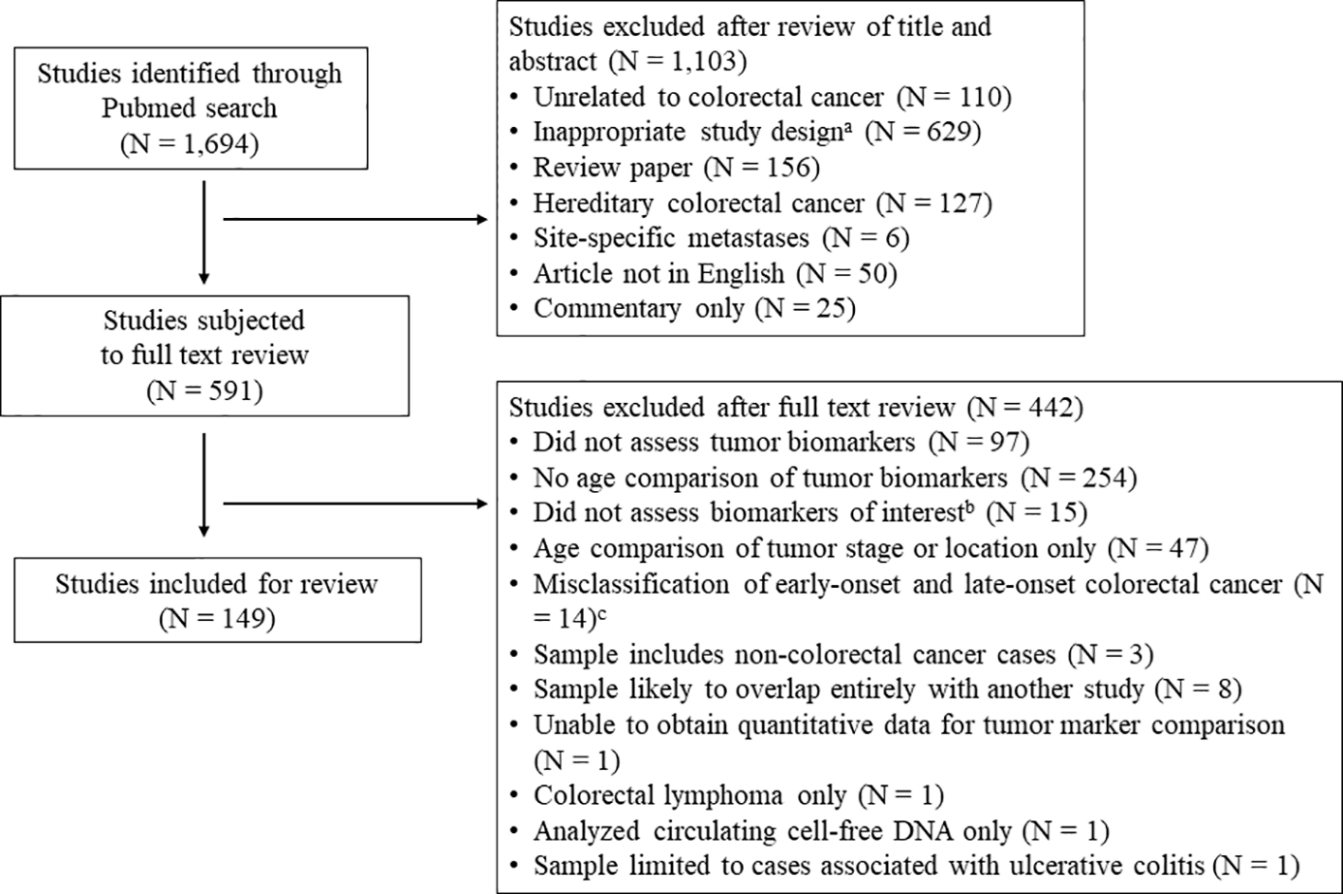
Literature review flowchart. ^a^ Inappropriate study design includes studies concerning colorectal cancer incidence, colonoscopy or other colorectal cancer screening, population level summary statistics for colorectal cancer, and studies of colorectal cancer in model organisms or *in vitro* studies. ^b^ Markers of interest include oncogene mutations in *KRAS*, *NRAS*, *BRAF*, *PIK3CA*, *PTEN*, *TP53*, *APC*, and *HER2*; histological phenotypes including high-grade tumors and mucinous or signet ring histology; molecular carcinogenesis pathways including microsatellite instability and the CpG island methylator phenotype (CIMP); and novel tumor prognostic phenotypes including immune markers in the tumor microenvironment and the consensus molecular subtypes.^c^ Studies where late-onset colorectal cancer was defined as ≥ 40 years at diagnosis (or younger), or early-onset CRC was defined as ≤ 60 years at diagnosis (or older).

Articles for this systematic review were identified utilizing a Pubmed search incorporating PRISMA guidelines (16). Given the wide breadth of the topic and the limited number of relevant articles published prior to 2013, the search was limited to peer-reviewed, original research articles published in English from the last 10 years (April 2013 – April 2023), with relevant keywords and medical subject headings included in the title and/or abstract. The literature review was repeated in January 2024 to identify recently published articles. Specific biomarker terms to include in the literature search were identified from prior reviews, and the search terms “biomark*”, “mark*”, and “character*” were included to capture potentially novel prognostic markers. All search terms included for the literature review are displayed in **Supplementary Table S1**. Manuscripts were included that reported the prevalence of prognostic biomarkers in CRC tumors separately for early- vs. late-onset disease. Articles were excluded if the prevalence of tumor clinicopathological or molecular biomarkers were not provided for participants with CRC (see **Figure 1** flowchart), or if there was no comparison between early- vs. late-onset CRC (or if the comparison was limited to tumor stage or location only). Articles were also excluded that described hereditary CRC only (e.g. Lynch syndrome), site-specific metastases, or included non-CRC cancers in the analysis samples. For the purposes of this analysis, early-onset disease was defined as CRC diagnosed prior to age 50. To avoid misclassification of early- and late-onset CRC, we excluded papers where late-onset CRC was defined as ≥ 40 years at diagnosis or younger, or where early-onset CRC was defined as ≤ 60 years at diagnosis or older. Lastly, to limit sample overlap where possible, we excluded studies if there was evidence of complete overlap in sample and markers reported with a previously published study, or if a study reported the same outcome in a subsample of a previous study.

The systematic review and meta-analysis was limited to the following markers that have been shown associations with CRC survival and/or therapeutic response in CRC: oncogene mutations in *KRAS* (17–20), *NRAS* (17, 21, 22), *BRAF* (17, 19, 23, 24), *PIK3CA* (17, 25, 26), *PTEN* (27, 28), *TP53* (29), *APC* (30, 31), and *HER2* amplifications (32–34); histological phenotypes including high-grade tumors (35, 36) and mucinous (37, 38) or signet ring histology (38, 39); molecular carcinogenesis pathways including MSI (40) and the CpG island methylator phenotype (CIMP) (41); and novel tumor prognostic phenotypes including immune markers (42, 43) in the tumor microenvironment and the consensus molecular subtypes (9, 44). Because it is well-established that early-onset CRC is associated with advanced tumor summary stage at diagnosis and rectal tumor location, these markers are not summarized in this review. The literature review was completed by two authors (T.L. and L.P) independently. Disagreements between reviewers were resolved by further review of the manuscript to determine whether the study included a comparison of tumor markers of interest between early- and late-onset CRC. The final decision to include a manuscript was made by the lead author. In total, 1,694 articles were identified from the literature search and 149 were eligible for review (**Figure 1**). For each study, the potential for bias was evaluated by the lead author using the Newcastle-Ottawa Scale adapted for cross-sectional studies (45). Pre-registration of the systematic review protocol was not performed.

### 2.2 Meta-analysis

From each eligible study, the number of mutant and wild-type tumors for each marker in early- and late-onset CRC was extracted by the lead author. Data extraction was completed in duplicate, and the results from the two extractions were compared to identify any errors or inconsistencies in the sample sizes, which were subsequently revised after further review of the original article. If these data were not available from the manuscript, sample sizes were requested from the corresponding author. One study was excluded for which we were unable to obtain the necessary sample sizes from each group (46). When necessary, sample sizes for separate age groups were combined to create a single category for early-onset and late-onset CRC. For most studies, age 45 or 50 at diagnosis was utilized as the threshold to distinguish early- vs. late-onset CRC, although occasionally other classifications were employed (see **Supplementary S2**). For each study, sample characteristics including overall sample size, country, tumor stage, sex, or other distinguishing features were also extracted. For each marker, an odds ratio (OR) and 95% confidence interval (CI) were calculated using a standard equation (47). For mutations in oncogenes *KRAS, NRAS, BRAF, PIK3CA, PTEN, TP53*, and *APC*, as well as MSI status and histological subtypes, meta-analyses were completed to compare the prevalence in tumors from early- vs. late-onset CRC. Due to the wide variety of immune markers that have been reported, a meta-analysis was not attempted for the comparison of immune phenotypes in the tumor microenvironment. For each marker that was meta-analyzed, a pooled OR with 95% CI was obtained from a random effects model via inverse variance weighting. The random effects model was selected *a priori*, as between-study heterogeneity is plausible given variability in the definition of early-onset CRC, as well as differences in tumor location, race, nationality and stage between studies. The random effects meta-analysis is capable of providing unbiased estimates in the presence of heterogeneity and will generally provide more conservative estimates than the fixed-effects model (which assumes no between-study heterogeneity) (48). Heterogeneity was determined via the Cochrane’s Q statistic and the I^2^ statistic. Significant heterogeneity was defined as P <.05 for Cochrane’s Q or I^2^ ≥ 50%. To determine whether the meta-analysis estimates were influenced by a single study, a ‘leave-one-out’ sensitivity analysis was conducted for each marker. Because Lynch syndrome may influence the prevalence of tumor markers for individuals with early-onset CRC, a second sensitivity analysis was completed to limit the analysis to studies that specifically excluded individuals with Lynch syndrome or family history of CRC, or that restricted the sample to microsatellite stable tumors. All statistical tests were two-sided, with statistical significance defined using a threshold of P <.05. All meta-analyses were completed using Review Manager 5.4.1 from Cochrane.

## 3 Results

In total, 149 articles were reviewed that compared the prevalence of clinicopathological tumor markers in early- vs. late-onset CRC. All meta-analysis results are summarized in **Table 1**. Sample characteristics and references for all included studies are presented in **Supplementary Table S2**. Results of the bias assessment utilizing the Newcastle-Ottawa Scale are presented in **Supplementary Table S4**.

**Table 1:** Summary of meta-analysis results showing associations between early-onset colorectal cancer and the prevalence of tumor markers, compared to late-onset colorectal cancer.

	All studies (N = 150)			Studies that excluded individuals with Lynch syndrome ^a^ (N = 50)		
Marker	Number of studies	OR (95% CI)	P-value	Number of studies	OR (95% CI)	P-value
*KRAS* mutation	54	0.91 (0.85-0.98)	.01	19	0.87 (0.80-0.95)	.002
*BRAF* mutation	54	0.63 (0.51-0.78)	<.001	17	0.77 (0.64-0.92)	.004
*NRAS* mutation	20	0.88 (0.78-1.00)	.06	6	0.89 (0.70-1.13)	.33
*APC* mutation	19	0.70 (0.58-0.84)	<.001	7	0.81 (0.67-0.97)	.02
*TP53* mutation	20	1.34 (1.24-1.45)	<.001	8	1.40 (1.32-1.48)	<.001
*PTEN* mutation	8	1.68 (1.04-2.73)	.04	3	2.81 (0.56-14.18)	.21
*PIK3CA* mutation	21	0.95 (0.86-1.05)	.29	8	0.88 (0.78-0.99)	.03
*HER2* amplification	4	1.64 (0.86-3.14)	.13	0	N/A	N/A
CIMP status	10	0.24 (0.10-0.57)	.001	7	0.41 (0.21-0.79)	.007
MSI status	64	1.31 (1.11-1.56)	.002	20	1.37 (0.91-2.07)	.13
High tumor grade	87	1.20 (1.15-1.25)	<.001	33	1.34 (1.15-1.57)	<.001
Mucinous histology	57	1.22 (1.16-1.27)	<.001	20	1.51 (1.23-1.84)	<.001
Signet ring histology	44	2.32 (2.08-2.57)	<.001	9	2.70 (1.77-4.14)	<.001

Data presented as odds ratio (95% confidence interval).

CI, confidence interval; CIMP, CpG island methylator phenotype; MSI, microsatellite instability; OR, odds ratio.

a Includes studies that excluded individuals with Lynch syndrome, all hereditary syndromes, microsatellite instability, or family history of colorectal cancer.N/A indicates not applicable.

### 3.1 Oncogene mutations

**Figure 2:**
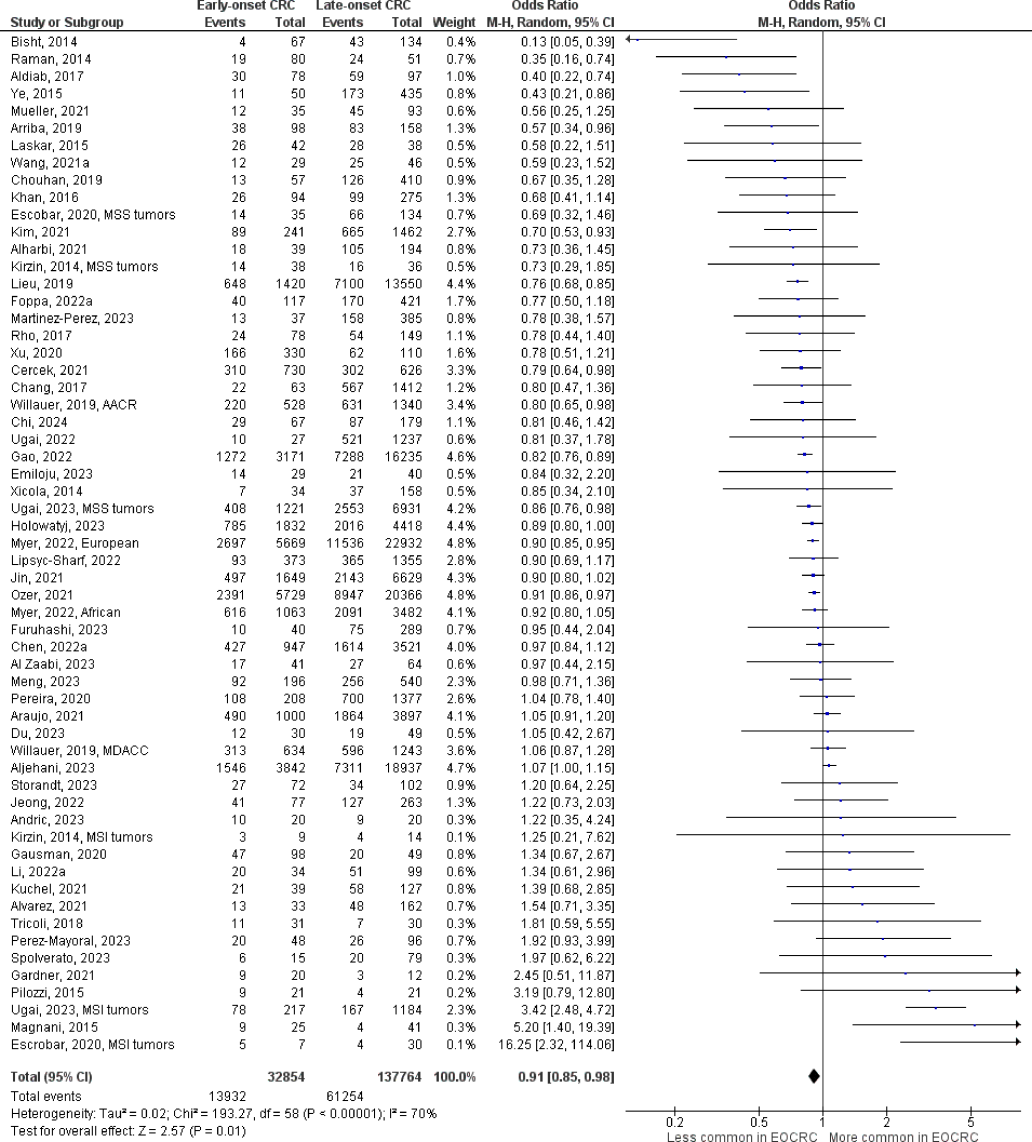
Odds ratios for *KRAS* mutation in early-onset CRC. Data presented as odds ratios (95% confidence interval) for *KRAS* mutation in early-onset relative to late-onset colorectal cancer. The pooled odds ratio is obtained via a random effects model using inverse variance weighting. AACR, American Association for Cancer Research; MDACC, MD Anderson Cancer Center; MSI, microsatellite instability; MSS, microsatellite stable.

**Figure 3:**
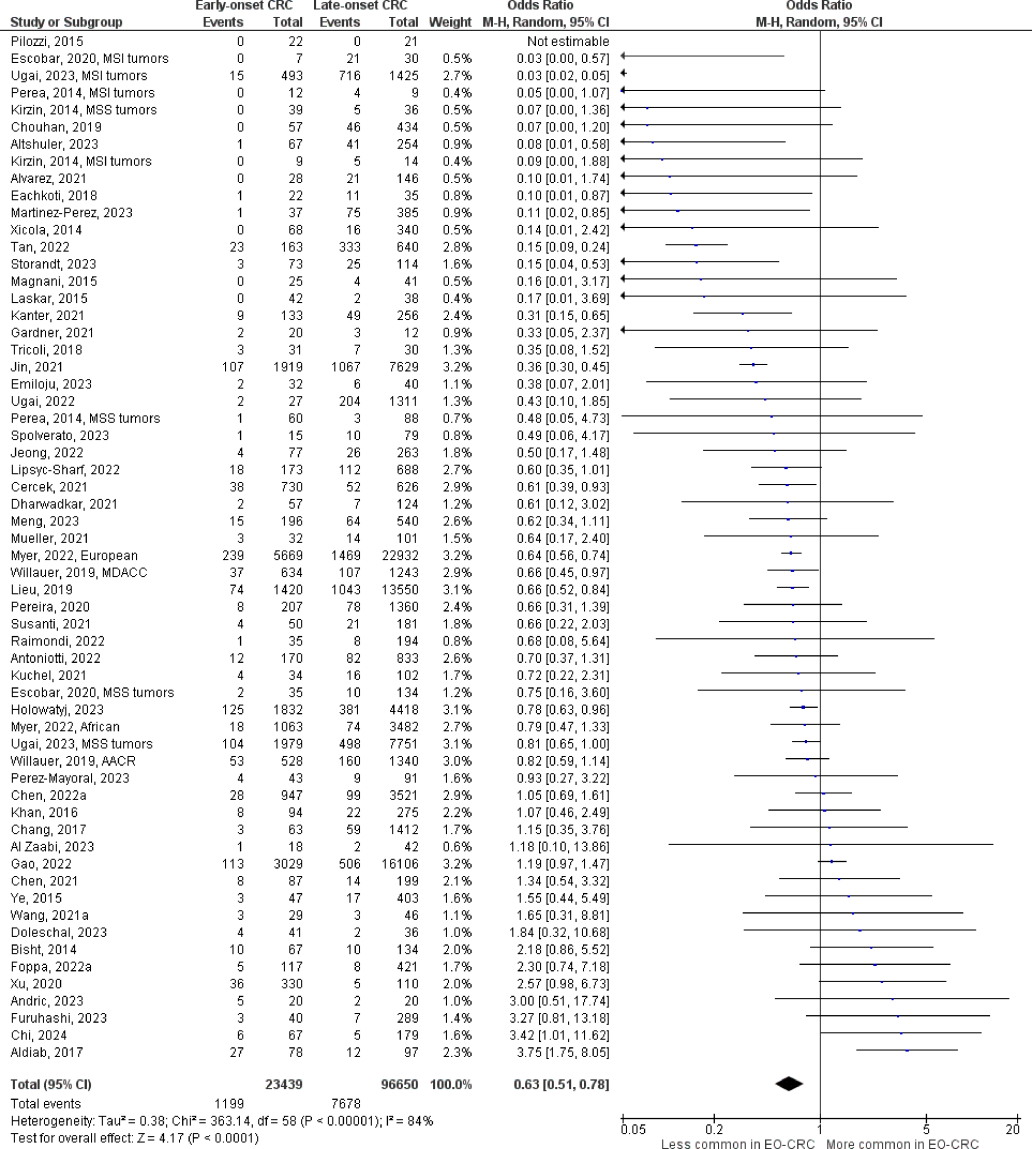
Odds ratios for *BRAF* mutation in early-onset CRC. Data presented as odds ratios (95% confidence interval) for *BRAF* mutation in early-onset relative to late-onset colorectal cancer. The pooled odds ratio is obtained via a random effects model using inverse variance weighting. AACR, American Association for Cancer Research; MDACC, MD Anderson Cancer Center; MSI, microsatellite instability; MSS, microsatellite stable.

**Figure 4:**
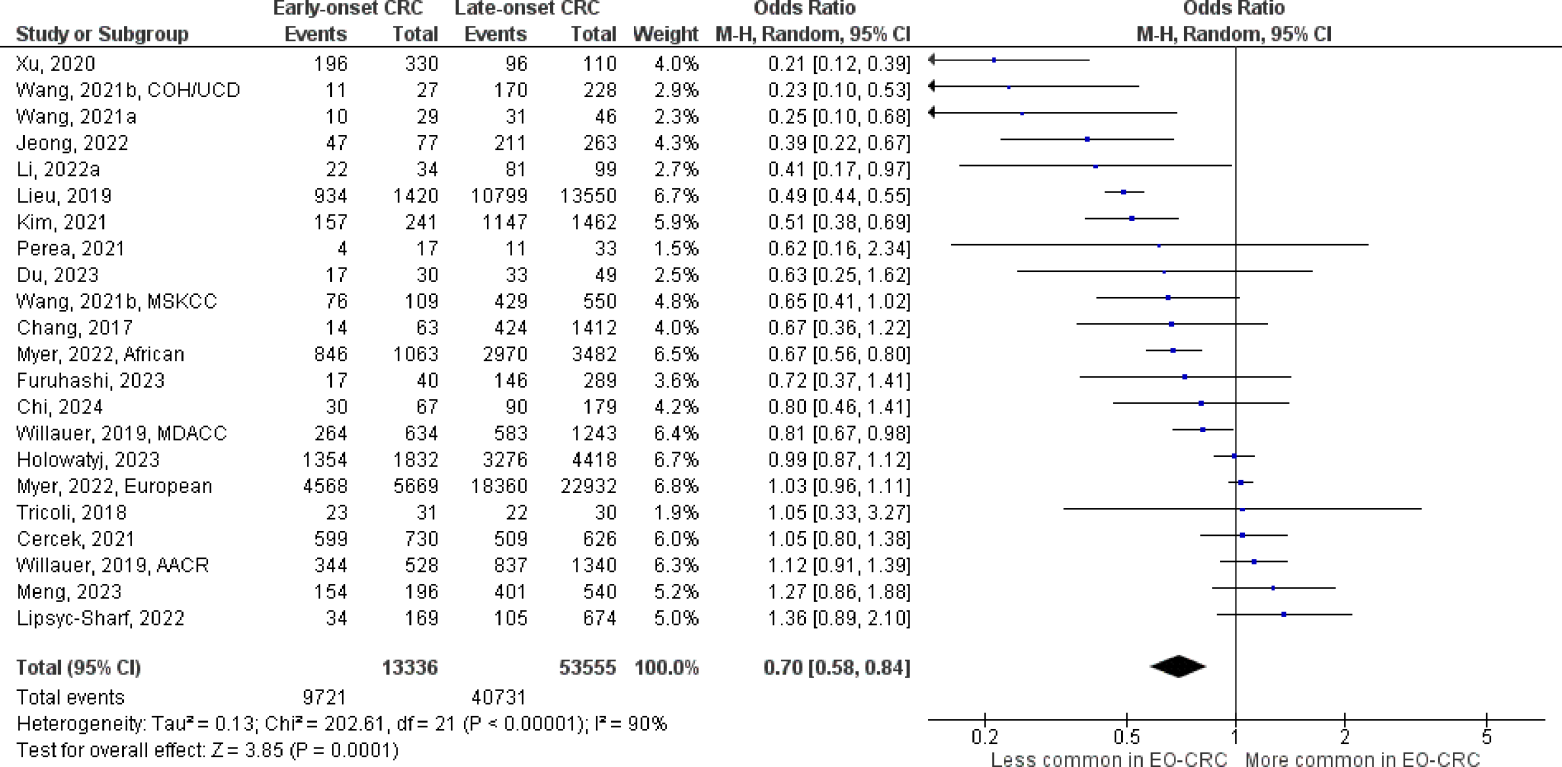
Odds ratios for *APC* mutation in early-onset colorectal cancer. Data presented as odds ratios (95% confidence interval) for *APC* mutation in early-onset relative to late-onset colorectal cancer. The pooled odds ratio is obtained via a random effects model using inverse variance weighting. AACR, American Association for Cancer Research; COH, City of Hope National Medical Center; CI, confidence interval; EO-CRC, early-onset colorectal cancer; MDACC, MD Anderson Cancer Center; MSKCC, Memorial Sloan Kettering Cancer Center; UCD, University of California, Davis.

**Figure 5:**
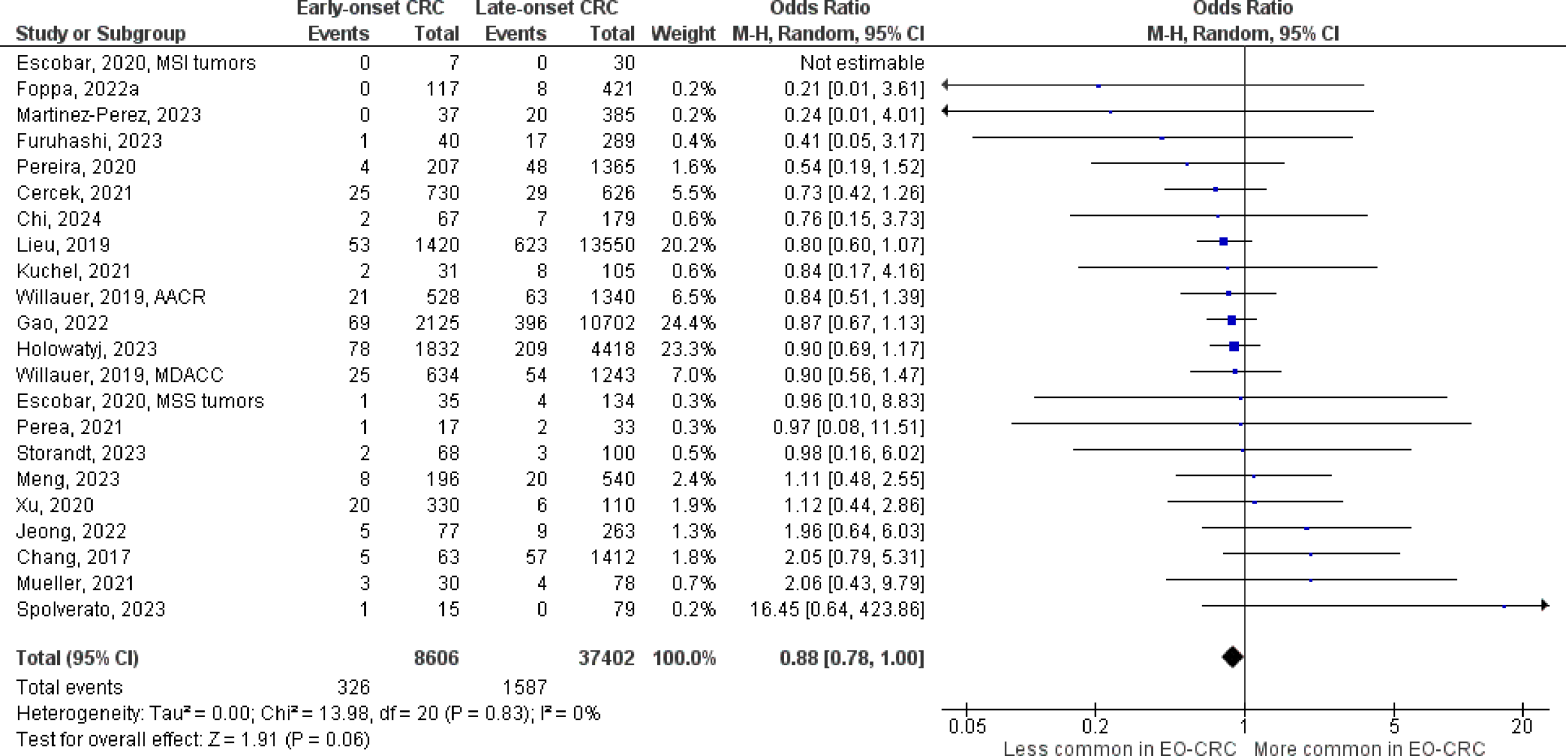
Odds ratios for *NRAS* mutation in early-onset colorectal cancer. Data presented as odds ratios (95% confidence interval) for *NRAS* mutation in early-onset relative to late-onset colorectal cancer. The pooled odds ratio is obtained via a random effects model using inverse variance weighting. AACR, American Association for Cancer Research; CI, confidence interval; EO-CRC, early-onset colorectal cancer; MDACC, MD Anderson Cancer Center.

**Figure 6:**
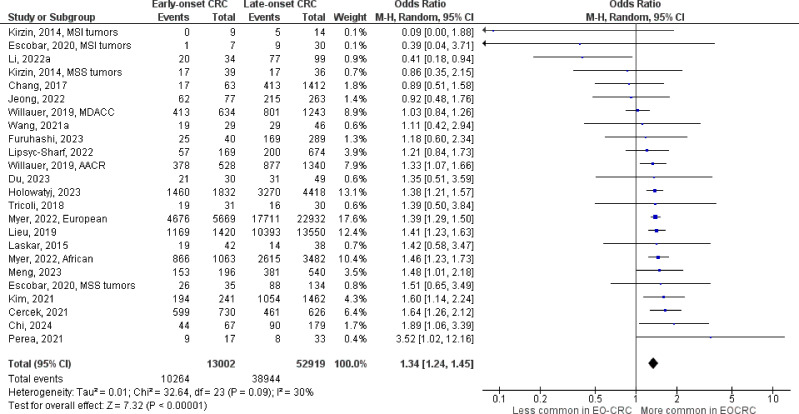
Odds ratios for *TP53* mutation in early-onset colorectal cancer. Data presented as odds ratios (95% confidence interval) for *TP53* mutation in early-onset relative to late-onset colorectal cancer. The pooled odds ratio is obtained via a random effects model using inverse variance weighting. AACR, American Association for Cancer Research; CI, confidence interval; EO-CRC, early-onset colorectal cancer; MDACC, MD Anderson Cancer Center; MSI, microsatellite instability; MSS, microsatellite stability.

**Figure 7:**
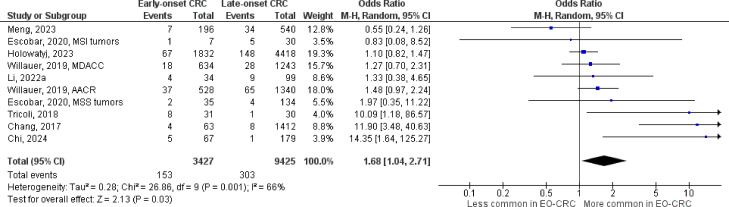
Odds ratios for *PTEN* mutation in early-onset colorectal cancer. Data presented as odds ratios (95% confidence interval) for *PTEN* mutation in early-onset relative to late-onset colorectal cancer. The pooled odds ratio is obtained via a random effects model using inverse variance weighting. AACR, American Association for Cancer Research; CI, confidence interval; EO-CRC, early-onset colorectal cancer; MDACC, MD Anderson Cancer Center.

The number of studies identified for the following markers is as follows: *KRAS* mutation (49); *BRAF* mutation (49); *NRAS* mutation (20); *PIK3CA* mutation (21); *PTEN* mutation (8); *HER2* amplifications (5); *APC* mutation (19); *TP53* mutation (20). For early-onset CRC, there is evidence for a significantly lower prevalence of mutations in *KRAS* (**Figure 2**, OR 0.91, 95% CI 0.85-0.98), *BRAF* (**Figure 3**, OR 0.63, 95% CI 0.51-0.78) and *APC* (**Figure 4**, OR 0.70, 95% CI 0.58-0.84) compared to late-onset CRC. Early-onset CRC was associated with non-significantly lower prevalence of mutations in *NRAS* (**Figure 5**, OR 0.88, 95% CI 0.78-1.00, p = .06). Conversely, early-onset CRC is associated with a higher prevalence of mutations in *TP53* (**Figure 6**, OR 1.34, 95% CI 1.24-1.45) and *PTEN* (**Figure 7**, OR 1.68, 95% CI 1.04-2.73). There was no significant difference in the prevalence of *PIK3CA* mutations (**Supplementary Figure S1**, OR 0.95, 95% CI 0.86-1.05), or *HER2* amplifications (**Supplementary Figure S2**, OR 1.64, 95% CI 0.86-3.14). Significant inter-study heterogeneity was observed for mutations in *KRAS*, *BRAF*, *PTEN*, and *APC*. Hazard ratios for oncogene mutations were stable in the leave-one-out sensitivity analysis (**Supplementary Table S3**), although the association for *NRAS* and *PTEN* mutations did not always reach statistical significance.

Fifty studies were identified that specifically excluded individuals with Lynch syndrome or family history of CRC, or that restricted the analysis to individuals with microsatellite stable tumors (**Table 1**; **Supplementary Table S2**). Compared to the full analysis, the association between early-onset CRC and *BRAF* (OR 0.77, 95% CI 0.64-0.92) and *APC* mutations (OR 0.81, 95% CI 0.67-0.97) were attenuated but remained statistically significant, while the associations with *KRAS*, *NRAS*, and *TP53* mutations were similar. Further, an inverse association between early-onset CRC and *PIK3CA* mutation was also observed (OR 0.88, 95% CI 0.78-0.99).

### 3.2 Molecular carcinogenesis pathways

There were 10 studies that compared the prevalence of CIMP-high status in early- vs. late-onset CRC, and 64 studies that compared MSI status. Individuals with early-onset CRC had significantly lower odds for CIMP-high tumors compared to individuals with late-onset disease (**Supplementary Figure S3**, OR 0.24, 0.10-0.57), but significantly higher odds for the MSI phenotype (**Supplementary Figure S4**, OR 1.31, 1.11-1.56). Significant heterogeneity was observed for both markers. Associations were stable in the leave-one-out sensitivity analysis (**Supplementary Table S3**), and after limiting the analysis to studies that excluded individuals with Lynch syndrome or family history of CRC (**Table 1**).

### 3.3 Histological characteristics

There were 86 studies that compared the prevalence of high-grade tumors (i.e. poorly differentiated or undifferentiated tumors) in early- vs. late-onset CRC, 57 studies that compared the prevalence of mucinous histology (or mucinous characteristics), and 44 studies that reported on signet ring cell carcinomas. In early-onset CRC, there was evidence for a significantly higher prevalence of high-grade (i.e., poorly differentiated) tumors (**Supplementary Figure S5**, OR 1.20, 95% CI 1.15-1.25), as well as mucinous tumors (**Supplementary Figure S6**, OR 1.22, 95% CI 1.16-1.27), and signet ring cell carcinomas (**Supplementary Figure S7**, OR 2.32, 2.08-2.57). Significant inter-study heterogeneity was observed for all histological markers. All associations were stable in the leave-one-out sensitivity analysis (**Supplementary Table S3**) and after limiting the analysis to studies that excluded individuals with Lynch syndrome or family history of CRC (**Table 1**).

### 3.4 Immune markers

There have been nine studies to investigate age differences in the immune cell populations of CRC tumors, with inconsistent results (49–57). Du et al. reported that Chinese patients with sporadic early-onset CRC showed significantly higher densities of multiple immune cell populations in the tumor microenvironment compared to patients with late-onset disease, including higher levels of B cells, CD4+ T cells, CD8+ T cells, neutrophils, macrophages, and dendritic cells (50). By contrast, Ugai et al. reported no significant differences in the populations of T cells, macrophages, and other myeloid cells in participants with early- vs. late-onset CRC from the Nurses’ Health Study and Health Professionals Follow-up Study (51). In a small study of 14 tumors utilizing single cell RNA sequencing, Li et al. reported that early-onset CRC was associated with lower levels of effector CD8+ T cells and antigen-presentation in the tumor microenvironment, but higher levels of naïve CD8+ T cells and immunosuppressive regulatory T cells compared to individuals with late-onset disease, suggesting an impaired anti-tumor immune response for early-onset CRC (54). Because MSI status may influence the anti-tumor immune response, recent studies have examined associations between early-onset CRC and tumor lymphocyte populations in samples limited to microsatellite stable tumors, or after careful exclusion of participants with Lynch syndrome (56, 57). In a matched analysis of microsatellite stable tumors, Lu et al. (2023) reported that there was no significant differences between early- and late-onset CRC for the infiltration of 22 different lymphocyte populations in the tumor microenvironment (57). Likewise, Andric et al. found no significant difference for five lymphocyte populations (total T cells, conventional CD4+ and CD8+ T cells, regulatory T cells, and γδ T cells) in a matched sample limited to cases of sporadic CRC (56). Other studies have reported no significant differences between early and late-onset CRC for the density of total tumor infiltrating lymphocytes (53, 55).

### 3.5 The consensus molecular subtypes

There have been six studies to determine the distribution of consensus molecular subtypes (CMS) for CRC by age at diagnosis (50, 57–61). Utilizing tumor tissues samples from 626 individuals diagnosed with CRC from The Cancer Genome Atlas and MD Anderson Cancer Center, Willauer reported that the CMS1 subtype was more common among patients aged 30-39 years at diagnosis (46%) compared to older participants, while the CMS4 subtype was less common (13%) (58). Conversely, in a smaller study from the Nanjing Colorectal Cancer Cohort, Du et al. reported a higher prevalence of the CMS4 subtype in early- vs. late-onset CRC (36.7% vs. 12.2%, respectively), although the comparison between age groups did not reach statistical significance (50). Recent results, including from a small sample of South Korean participants (59) and additional analyses of The Cancer Genome Atlas (60, 61) did not show any significant association between early-onset tumors and the distribution of consensus molecular subtypes.

## 4 Discussion

Sporadic early-onset CRC is a significant public health concern, increasing by 2-3% per year in the U.S. since 1990 (3, 62). Early-onset CRC is more often diagnosed at advanced stages compared to late-onset disease (63, 64). However, there is inconsistent evidence that survival varies between early- and late-onset CRC (65, 66), complicated by reports that younger patients receive more aggressive systemic treatment (67–69). Thus, international guidelines do not endorse separate treatment recommendations for early-onset disease (70). Investigating the associations between early-onset tumors and molecular and histological characteristics, and novel tumor markers including immune cell populations, may help to guide the development of therapies that benefit early-onset CRC. Further, highlighting associations between early-onset CRC and tumor markers may aid in the design of clinical trials for targeted therapies. To the authors’ knowledge, this is the first comprehensive systematic review and meta-analysis of tumor prognostic and predictive markers in early-onset CRC. We found that early-onset CRC was associated with a lower prevalence of oncogene mutations in *KRAS*, *BRAF*, *NRAS*, and *APC*, but a higher prevalence of *TP53* and *PTEN* mutations and adverse histologic subtypes, with inconsistent associations for immune cell populations and the consensus molecular subtypes.

*KRAS*, *BRAF*, and *NRAS* encode proteins that act downstream of the epidermal growth factor receptor (EGFR) and activate Mek/Erk signaling (21, 71). Mutations in these oncogenes are negative predictive markers for EGFR inhibition in metastatic CRC (17, 18) and are associated with inferior survival outcomes across tumor stage (19, 20, 23, 72), including for early-onset CRC (73–75). Early-onset CRC is associated with a lower prevalence of mutations in these genes compared to late-onset disease, indicating that individuals with metastatic early-onset CRC may be more likely to benefit from EGFR inhibition. Notably, the association with *NRAS* mutations was not statistically significant, which may be due to the scarcity of this marker (76). Further, the association with *BRAF* mutation was attenuated but still statistically significant in studies that excluded individuals with Lynch syndrome, who are less likely to have *BRAF* mutations compared to sporadic disease (77). Further, this sensitivity analysis revealed an inverse association with *PIK3CA* mutation, which has also been linked to higher risk for mortality and resistance to EGFR inhibition (17, 78). Conversely, early-onset CRC was associated with a *higher* proportion of mutations in tumor suppressor *PTEN*, which encodes a lipid-phosphatase that suppresses the activity of PI3k/Akt/mTOR signaling and interacts with the EGFR pathway (27). Loss of PTEN activity has been linked to resistance to EGFR inhibition in metastatic CRC (79) but is not currently used in clinical decision making. Pharmaceutical therapies to restore normal PTEN activity are under development but have not been evaluated in CRC. Early-onset CRC was associated with a significantly higher prevalence of *TP53* mutations, which cause loss of p53 tumor suppressor activity and pro-tumorigenic gain of function effects that accelerate cell proliferation, angiogenesis, and metastasis (80). *TP53* mutations are found in approximately 60% of tumors and may promote resistance to EGFR inhibitors and chemotherapies that rely on wild type p53 to induce cellular apoptosis (e.g. 5-fluorouracil and Oxaliplatin) (29). Consequently, targeted therapies to restore wild type p53 activity or degrade mutant p53, or to inhibit downstream effector pathways, are currently being investigated in clinical trials (81). Potentially, individuals with early-onset CRC may be more likely to benefit from treatments that inhibit pro-tumorigenic p53 activity and should be targeted for enrollment in these trials.

Early-onset CRC was associated with a lower prevalence of *APC* mutation, a key driver of the canonical adenoma-carcinoma pathway (82). *APC* mutations are present in approximately 80% of CRC tumors (11, 12, 14), and recent evidence indicates that *APC*-mutant tumors are associated with extended overall and progression-free survival compared to wild type (30, 31) (5). Notably, the association with *APC* mutation was attenuated but still statistically significant when limiting the analysis to studies that excluded individuals with Lynch syndrome, or that included microsatellite stable tumors only. Individuals with early-onset CRC had a higher prevalence of MSI, defined by a high density of somatic mutations in short, non-coding sequences caused by defects in DNA mismatch repair (40). MSI is associated with lower risk for overall mortality and distant metastases compared to microsatellite stable tumors, including in early-onset CRC (75). Further, MSI tumors secrete truncated proteins that trigger an anti-tumor immune response (83), and consequently MSI is a positive predictor for response to immune checkpoint inhibitors (83). Our findings therefore highlight the importance of MSI testing for individuals younger than 50, in accordance with clinical guidelines (70). Unexpectedly, the association between early-onset CRC and MSI status was modestly strengthened in studies that excluded individuals with known Lynch syndrome, which causes tumors with MSI (84). Because a significant proportion of individuals with Lynch syndrome may be unaware of the condition (85), it is possible that the exclusion of Lynch syndrome was incomplete in some studies. Early-onset CRC was associated with a lower prevalence of the CpG island methylator phenotype (CIMP), characterized by methylation and inactivation of tumor-suppressor genes (86). Although CIMP has been linked to poor prognosis in multiple studies, it currently has limited value as a prognostic marker due to a lack of standardized assessment and competing effects of MSI and *BRAF* mutation, which are associated with CIMP (41).

We also found that early-onset CRC is associated with higher odds for tumors with more aggressive histological features, including poorly differentiated tumors, mucinous carcinomas, and signet ring cell carcinomas (38, 87). The association with signet ring features was especially pronounced (OR [95% CI]: 2.32 [2.08-2.57]). Although signet ring carcinomas comprise only 1% of CRC tumors (39), this feature is present in 2-3% of early-onset tumors. A recent meta-analysis showed that signet ring carcinomas were associated with significantly higher risk for overall mortality and recurrence compared to conventional adenocarcinomas (88). Results were similar for mucinous tumors, which comprise approximately 10-15% of CRCs (89). The associations between histological subtypes and colorectal cancer mortality, especially poorly differentiated tumors and signet ring carcinomas, have been validated in early-onset CRC (90–93). Currently, there are no treatments that specifically target mucinous or signet ring cell carcinomas and treatment guidelines do not distinguish between histological subtypes (70).

The observed associations between early-onset CRC and certain histological and molecular tumor characteristics may be explained in part by differences in tumor location (94). Approximately 30% of early-onset tumors are located in the rectum, versus 20% of late-onset tumors (64, 95). *KRAS, BRAF, PIK3CA*, and *NRAS* mutations are enriched in proximal tumors (96, 97) while *TP53* mutations are enriched in rectal tumors (98). Notably, studies that were limited to individuals with tumors in the distal colon or rectum have not shown a consistent association between early-onset CRC and the presence of oncogene mutations (46, 55, 56, 99–102). For example, a study with more than 1,000 distal and rectal tumors showed no significant age difference in *KRA*S, *BRAF, NRAS*, *PIK3CA*, *TP53*, or *APC* mutations (46). Conversely, in a large-scale analysis with detailed stratification by tumor location, Ugai et al. found that early-onset CRC had a lower prevalence of *BRAF* mutations for all tumor sites except the sigmoid colon and rectum (103). Notably, aggressive histological subtypes are overrepresented in the proximal colon (104), and consequently the association with early-onset CRC is not explained by differences in tumor location.

We found inconsistent evidence linking early-onset CRC to differences in ‘novel’ tumor prognostic and predictive markers including populations of immune cells in the tumor microenvironment (8). A recent meta-analysis demonstrated that a higher density of tumor infiltrating lymphocytes was associated with reduced overall mortality among 20,015 individuals with CRC (HR [95% CI]: 0.65 [0.54-0.77]) (42), while others have shown that an ‘immunoscore’ encompassing cytotoxic T cells and CD3+ cells was a superior prognostic marker compared to the tumor stage (105, 106). Currently, the association between early-onset CRC and the anti-tumor immune response has been inconsistent (48–50, 52, 53, 55, 56, 58). Notably, higher rates of MSI in early-onset CRC due to Lynch syndrome may obscure associations with immune markers in sporadic disease, as MSI tumors trigger a robust anti-tumor immune response (83). Studies limited to microsatellite stable tumors or that carefully excluded participants with hereditary syndromes have tended to show no significant differences in immune cell populations between early- and late-onset CRC (51, 56, 57). Likewise, there is currently no consistent evidence that the distribution of consensus molecular subtypes differs between early- and late-onset CRC, with most studies reporting null findings (50, 57, 59–61). The consensus molecular subtypes have shown to be a robust predictor of mortality outcomes independent of tumor stage (107), but to the authors’ knowledge have not been validated specifically in early-onset CRC. Further, the identification of novel molecular subtypes in early-onset CRC based on tumor gene expression is an area for future research.

Strengths of this study include the comprehensive nature of the search strategy, as we were able to summarize the evidence for age-related differences in the prevalence of established tumor prognostic markers as well as emerging markers including immune cell populations in the tumor microenvironment and the consensus molecular subtypes. Further, the large number of studies identified for most markers allowed for relatively precise estimates of the association with early-onset CRC. Lastly, to better understand the associations between early-onset CRC and tumor markers in *sporadic* disease, we completed a sensitivity analysis limited to studies that excluded individuals with known Lynch syndrome (or family history of CRC). This analysis is also attended by several limitations. Due to the breadth of the review, our literature search was limited to original research studies published within the last ten years in Pubmed. Consequently, it is possible that a relevant study was missed. However, this is unlikely to be a significant limitation given the paucity of large tumor genomic studies published prior to 2013 and the comprehensive nature of our search strategy. Further, there was evidence for significant heterogeneity in the estimates for most tumor markers, but we were unable to investigate underlying sources of inter-study heterogeneity because the prevalence of tumor prognostic markers was rarely presented in subgroups defined by tumor location, tumor stage, or MSI status. Between-study differences in the definitions of early- and late-onset CRC may also have contributed to heterogeneity, although we excluded studies where misclassification of early-onset CRC was apparent. Lastly, although we attempted to control for bias by performing a sensitivity analysis limited to studies that accounted for Lynch syndrome in the study design, it is possible that residual confounding by hereditary conditions or differences in tumor location may have biased the results.

## 5 Conclusions

In summary, early-onset CRC was associated with a lower prevalence of mutations in several oncogenes linked to mortality and poor therapeutic response, including *KRAS*, *BRAF*, and *NRAS* compared to individuals with late-onset disease. Conversely, early-onset disease was associated with a higher prevalence of potentially harmful mutations in *TP53* and *PTEN*, as well as aggressive histological subtypes including mucinous and signet ring cell carcinomas. In part, these associations may reflect the higher prevalence of rectal tumors in early-onset CRC and the effect of hereditary syndromes on tumor markers. Given these findings and the alarming rise in the incidence of early-onset CRC, it is essential that clinical trials for targeted therapies enroll sufficient numbers of individuals with early-onset disease to evaluate their efficacy in this subgroup. Additional research is required to clarify the relationships with novel tumor characteristics including immune markers and to identify molecular subtypes specific to early-onset CRC that can inform treatment and prognosis.

## Data availability statement

The original contributions presented in the study are included in the article/**Supplementary Material**. Further inquiries can be directed to the corresponding author.

## Author contributions

TL: Writing – review & editing, Writing – original draft, Visualization, Methodology, Investigation, Formal analysis, Data curation. LP: Writing – review & editing, Writing – original draft, Investigation, Data curation. SW: Writing – review & editing, Supervision, Resources, Project administration, Methodology, Funding acquisition, Conceptualization.

## References

[1] Siegel RL Wagle NS Cercek A Smith RA Jemal A . Colorectal cancer statistics, 2023. CA Cancer J Clin. (2023). 73(3):233–54. doi: 10.3322/caac.21772 36856579

[2] Weinberg BA Marshall JL . Colon cancer in young adults: trends and their implications. Curr Oncol Rep. (2019) 21:3. doi: 10.1007/s11912-019-0756-8 30659375

[3] Murphy CC Singal AG Baron JA Sandler RS . Decrease in incidence of young-onset colorectal cancer before recent increase. Gastroenterology. (2018) 155:1716–9. doi: 10.1053/j.gastro.2018.07.045 PMC6279567 30165046

[4] Done JZ Fang SH . Young-onset colorectal cancer: A review. World J Gastrointest Oncol. (2021) 13:856–66. doi: 10.4251/wjgo.v13.i8.856 PMC8371519 34457191

[5] Ballester V Rashtak S Boardman L . Clinical and molecular features of young-onset colorectal cancer. World J Gastroenterol. (2016) 22:1736–44. doi: 10.3748/wjg.v22.i5.1736 PMC4724605 26855533

[6] Sagaert X Vanstapel A Verbeek S . Tumor heterogeneity in colorectal cancer: what do we know so far? Pathobiology. (2018) 85:72–84. doi: 10.1159/000486721 29414818

[7] Gonzalez-Pons M Cruz-Correa M . Colorectal cancer biomarkers: where are we now? BioMed Res Int. (2015) 2015:149014. doi: 10.1155/2015/149014 26106599 PMC4461726

[8] Bai Z Zhou Y Ye Z Xiong J Lan H Wang F . Tumor-infiltrating lymphocytes in colorectal cancer: the fundamental indication and application on immunotherapy. Front Immunol. (2021) 12:808964. doi: 10.3389/fimmu.2021.808964 35095898 PMC8795622

[9] Guinney J Dienstmann R Wang X de Reyniès A Schlicker A Soneson C . The consensus molecular subtypes of colorectal cancer. Nat Med. (2015) 21:1350–6. doi: 10.1038/nm.3967 PMC4636487 26457759

[10] Lech G Słotwiński R Słodkowski M Krasnodębski IW . Colorectal cancer tumour markers and biomarkers: Recent therapeutic advances. World J Gastroenterol. (2016) 22:1745–55. doi: 10.3748/wjg.v22.i5.1745 PMC4724606 26855534

[11] Myer PA Lee JK Madison RW Pradhan K Newberg JY Isasi CR . The genomics of colorectal cancer in populations with african and european ancestry. Cancer Discovery. (2022) 12:1282–93. doi: 10.1158/2159-8290.CD-21-0813 PMC9169495 35176763

[12] Lieu CH Golemis EA Serebriiskii IG Newberg J Hemmerich A Connelly C . Comprehensive genomic landscapes in early and later onset colorectal cancer. Clin Cancer Res. (2019) 25:5852–8. doi: 10.1158/1078-0432.CCR-19-0899 PMC6774873 31243121

[13] Gao XH Li J Liu LJ Zheng NX Zheng K Mei Z . Trends, clinicopathological features, surgical treatment patterns and prognoses of early-onset versus late-onset colorectal cancer: A retrospective cohort study on 34067 patients managed from 2000 to 2021 in a Chinese tertiary center. Int J Surg. (2022) 104:106780. doi: 10.1016/j.ijsu.2022.106780 35850466

[14] Holowatyj AN Wen W Gibbs T Seagle HM Keller SR Edwards DRV . Racial/ethnic and sex differences in somatic cancer gene mutations among patients with early-onset colorectal cancer. Cancer Discovery. (2023) 13:570–9. doi: 10.1158/2159-8290.CD-22-0764 PMC10436779 36520636

[15] Venugopal A Carethers JM . Epidemiology and biology of early onset colorectal cancer. EXCLI J. (2022) 21:162–82. doi: 10.17179/excli2021-4456 PMC8859644 35221839

[16] Page MJ McKenzie JE Bossuyt PM Boutron I Hoffmann TC Mulrow CD . The PRISMA 2020 statement: an updated guideline for reporting systematic reviews. BMJ. (2021) 372:n71. doi: 10.1136/bmj.n71 33782057 PMC8005924

[17] Therkildsen C Bergmann TK Henrichsen-Schnack T Ladelund S Nilbert M . The predictive value of KRAS, NRAS, BRAF, PIK3CA and PTEN for anti-EGFR treatment in metastatic colorectal cancer: A systematic review and meta-analysis. Acta Oncol. (2014) 53:852–64. doi: 10.3109/0284186X.2014.895036 24666267

[18] Peeters M Price TJ Cervantes A Sobrero AF Ducreux M Hotko Y . Randomized phase III study of panitumumab with fluorouracil, leucovorin, and irinotecan (FOLFIRI) compared with FOLFIRI alone as second-line treatment in patients with metastatic colorectal cancer. J Clin Oncol. (2010) 28:4706–13. doi: 10.1200/JCO.2009.27.6055 20921462

[19] Formica V Sera F Cremolini C Riondino S Morelli C Arkenau HT . KRAS and BRAF mutations in stage II and III colon cancer: A systematic review and meta-analysis. J Natl Cancer Inst. (2022) 114:517–27. doi: 10.1093/jnci/djab190 PMC9002292 34542636

[20] Modest DP Ricard I Heinemann V Hegewisch-Becker S Schmiegel W Porschen R . Outcome according to KRAS-, NRAS- and BRAF-mutation as well as KRAS mutation variants: pooled analysis of five randomized trials in metastatic colorectal cancer by the AIO colorectal cancer study group. Ann Oncol. (2016) 27:1746–53. doi: 10.1093/annonc/mdw261 PMC4999563 27358379

[21] De Roock W Claes B Bernasconi D De Schutter J Biesmans B Fountzilas G . Effects of KRAS, BRAF, NRAS, and PIK3CA mutations on the efficacy of cetuximab plus chemotherapy in chemotherapy-refractory metastatic colorectal cancer: a retrospective consortium analysis. Lancet Oncol. (2010) 11:753–62. doi: 10.1016/S1470-2045(10)70130-3 20619739

[22] Schirripa M Cremolini C Loupakis F Morvillo M Bergamo F Zoratto F . Role of NRAS mutations as prognostic and predictive markers in metastatic colorectal cancer. Int J Cancer. (2015) 136:83–90. doi: 10.1002/ijc.v136.1 24806288

[23] Jones JC Renfro LA Al-Shamsi HO Schrock AB Rankin A Zhang BY . Non-V600 BRAF mutations define a clinically distinct molecular subtype of metastatic colorectal cancer. J Clin Oncol. (2017) 35:2624–30. doi: 10.1200/JCO.2016.71.4394 PMC5549454 28486044

[24] Yang ZY Wu XY Huang YF Di MY Zheng DY Chen JZ . Promising biomarkers for predicting the outcomes of patients with KRAS wild-type metastatic colorectal cancer treated with anti-epidermal growth factor receptor monoclonal antibodies: a systematic review with meta-analysis. Int J Cancer. (2013) 133:1914–25. doi: 10.1002/ijc.v133.8 23494461

[25] Tan ES Fan W Knepper TC Schell MJ Sahin IH Fleming JB . Prognostic and predictive value of PIK3CA mutations in metastatic colorectal cancer. Target Oncol. (2022) 17:483–92. doi: 10.1007/s11523-022-00898-7 35767139

[26] Wu S Gan Y Wang X Liu J Li M Tang Y . PIK3CA mutation is associated with poor survival among patients with metastatic colorectal cancer following anti-EGFR monoclonal antibody therapy: a meta-analysis. J Cancer Res Clin Oncol. (2013) 139:891–900. doi: 10.1007/s00432-013-1400-x 23435830 PMC11824679

[27] Salvatore L Calegari MA Loupakis F Fassan M Di Stefano B Bensi M . PTEN in colorectal cancer: shedding light on its role as predictor and target. Cancers (Basel). (2019) 11:E1765. doi: 10.3390/cancers11111765 PMC6896095 31717544

[28] Molinari F Frattini M . Functions and regulation of the PTEN gene in colorectal cancer. Front Oncol. (2013) 3:326. doi: 10.3389/fonc.2013.00326 24475377 PMC3893597

[29] Liebl MC Hofmann TG . The role of p53 signaling in colorectal cancer. Cancers (Basel). (2021) 13:2125. doi: 10.3390/cancers13092125 33924934 PMC8125348

[30] Wang C Ouyang C Cho M Ji J Sandhu J Goel A . Wild-type APC is associated with poor survival in metastatic microsatellite stable colorectal cancer. Oncologist. (2021) 26:208–14. doi: 10.1002/onco.13607 PMC7930429 33230914

[31] Jorissen RN Christie M Mouradov D Sakthianandeswaren A Li S Love C . Wild-type APC predicts poor prognosis in microsatellite-stable proximal colon cancer. Br J Cancer. (2015) 113:979–88. doi: 10.1038/bjc.2015.296 PMC4578087 26305864

[32] Park DI Kang MS Oh SJ Kim HJ Cho YK Sohn CI . HER-2/neu overexpression is an independent prognostic factor in colorectal cancer. Int J Colorectal Dis. (2007) 22:491–7. doi: 10.1007/s00384-006-0192-8 16947041

[33] Sawada K Nakamura Y Yamanaka T Kuboki Y Yamaguchi D Yuki S . Prognostic and predictive value of HER2 amplification in patients with metastatic colorectal cancer. Clin Colorectal Cancer. (2018) 17:198–205. doi: 10.1016/j.clcc.2018.05.006 29866615

[34] Sartore-Bianchi A Amatu A Porcu L Ghezzi S Lonardi S Leone F . HER2 positivity predicts unresponsiveness to EGFR-targeted treatment in metastatic colorectal cancer. Oncologist. (2019) 24:1395–402. doi: 10.1634/theoncologist.2018-0785 PMC6795149 30952821

[35] Marks KM West NP Morris E Quirke P . Clinicopathological, genomic and immunological factors in colorectal cancer prognosis. Br J Surg. (2018) 105:e99–109. doi: 10.1002/bjs.10756 29341159 PMC7938848

[36] Compton CC . Colorectal carcinoma: diagnostic, prognostic, and molecular features. Mod Pathol. (2003) 16:376–88. doi: 10.1097/01.MP.0000062859.46942.93 12692203

[37] Luo ZW Zhu MG Zhang ZQ Ye FJ Huang WH Luo XZ . Increased expression of Ki-67 is a poor prognostic marker for colorectal cancer patients: a meta analysis. BMC Cancer. (2019) 19:123. doi: 10.1186/s12885-019-5324-y 30727976 PMC6364416

[38] Nitsche U Zimmermann A Späth C Müller T Maak M Schuster T . Mucinous and signet-ring cell colorectal cancers differ from classical adenocarcinomas in tumor biology and prognosis. Ann Surg. (2013) 258:775–82. doi: 10.1097/SLA.0b013e3182a69f7e PMC3888475 23989057

[39] An Y Zhou J Lin G Wu H Cong L Li Y . Clinicopathological and molecular characteristics of colorectal signet ring cell carcinoma: A review. Pathol Oncol Res. (2021) 27:1609859. doi: 10.3389/pore.2021.1609859 34381313 PMC8351516

[40] Diao Z Han Y Chen Y Zhang R Li J . The clinical utility of microsatellite instability in colorectal cancer. Crit Rev Oncol Hematol. (2021) 157:103171. doi: 10.1016/j.critrevonc.2020.103171 33290824

[41] Rhee YY Kim KJ Kang GH . CpG island methylator phenotype-high colorectal cancers and their prognostic implications and relationships with the serrated neoplasia pathway. Gut Liver. (2017) 11:38–46. doi: 10.5009/gnl15535 27885175 PMC5221859

[42] Idos GE Kwok J Bonthala N Kysh L Gruber SB Qu C . The prognostic implications of tumor infiltrating lymphocytes in colorectal cancer: A systematic review and meta-analysis. Sci Rep. (2020) 10:3360. doi: 10.1038/s41598-020-60255-4 32099066 PMC7042281

[43] Ogino S Nosho K Irahara N Meyerhardt JA Baba Y Shima K . Lymphocytic reaction to colorectal cancer is associated with longer survival, independent of lymph node count, microsatellite instability, and CpG island methylator phenotype. Clin Cancer Res. (2009) 15:6412–20. doi: 10.1158/1078-0432.CCR-09-1438 PMC2771425 19825961

[44] Valenzuela G Canepa J Simonetti C Solo de Zaldívar L Marcelain K González-Montero J . Consensus molecular subtypes of colorectal cancer in clinical practice: A translational approach. World J Clin Oncol. (2021) 12:1000–8. doi: 10.5306/wjco.v12.i11.1000 PMC8641009 34909395

[45] Herzog R Álvarez-Pasquin MJ Díaz C Del Barrio JL Estrada JM Gil Á . Are healthcare workers’ intentions to vaccinate related to their knowledge, beliefs and attitudes? A systematic review. BMC Public Health. (2013) 13:154. doi: 10.1186/1471-2458-13-154 23421987 PMC3602084

[46] Puccini A Lenz HJ Marshall JL Arguello D Raghavan D Korn WM . Impact of patient age on molecular alterations of left-sided colorectal tumors. Oncologist. (2019) 24:319–26. doi: 10.1634/theoncologist.2018-0117 PMC6519749 30018131

[47] Tenny S Hoffman MR . Odds ratio. In: StatPearls. Treasure Island (FL): StatPearls Publishing. (2024) Available at: http://www.ncbi.nlm.nih.gov/books/NBK431098/. 28613750

[48] Riley RD Higgins JPT Deeks JJ . Interpretation of random effects meta-analyses. BMJ. (2011) 342:d549. doi: 10.1136/bmj.d549 21310794

[49] Wang MJ Ping J Li Y Adell G Arbman G Nodin B . The prognostic factors and multiple biomarkers in young patients with colorectal cancer. Sci Rep. (2015) 5:10645. doi: 10.1038/srep10645 26013439 PMC4445043

[50] Du M Gu D Xin J Peters U Song M Cai G . Integrated multi-omics approach to distinct molecular characterization and classification of early-onset colorectal cancer. Cell Rep Med. (2023) 4:100974. doi: 10.1016/j.xcrm.2023.100974 36921601 PMC10040411

[51] Ugai T Väyrynen JP Lau MC Borowsky J Akimoto N Väyrynen SA . Immune cell profiles in the tumor microenvironment of early-onset, intermediate-onset, and later-onset colorectal cancer. Cancer Immunol Immunother. (2022) 71:933–42. doi: 10.1007/s00262-021-03056-6 PMC8924022 34529108

[52] Gardner IH Siddharthan R Watson K Dewey E Ruhl R Khou S . A distinct innate immune signature of early onset colorectal cancer. Immunohorizons. (2021) 5:489–99. doi: 10.4049/immunohorizons.2000092 PMC8763397 34162701

[53] Irabor DO Oluwasola OA Ogunbiyi OJ Ogun OG Okolo CA Melas M . Microsatellite instability is common in colorectal cancer in native Nigerians. Anticancer Res. (2017) 37:2649–54. doi: 10.21873/anticanres 28476840

[54] Li GM Xiao GZ Qin PF Wan XY Fu YJ Zheng YH . Single-cell RNA sequencing reveals heterogeneity in the tumor microenvironment between young-onset and old-onset colorectal cancer. Biomolecules. (2022) 12:1860. doi: 10.3390/biom12121860 36551288 PMC9776336

[55] Pilozzi E Maresca C Duranti E Giustiniani MC Catalanotto C Lucarelli M . Left-sided early-onset vs late-onset colorectal carcinoma: histologic, clinical, and molecular differences. Am J Clin Pathol. (2015) 143:374–84. doi: 10.1309/AJCPNOC55IOLXFUD 25696795

[56] Andric F Al-Fairouzi A Wettergren Y Szeponik L Bexe-Lindskog E Cusack JC . Immune microenvironment in sporadic early-onset versus average-onset colorectal cancer. Cancers (Basel). (2023) 15:1457. doi: 10.3390/cancers15051457 36900249 PMC10001362

[57] Lu C Zhang X Schardey J Wirth U Heinrich K Massiminio L . Molecular characteristics of microsatellite stable early-onset colorectal cancer as predictors of prognosis and immunotherapeutic response. NPJ Precis Oncol. (2023) 7:63. doi: 10.1038/s41698-023-00414-8 37393364 PMC10314951

[58] Willauer AN Liu Y Pereira AAL Lam M Morris JS Raghav KPS . Clinical and molecular characterization of early-onset colorectal cancer. Cancer. (2019) 125:2002–10. doi: 10.1002/cncr.31994 PMC6583775 30854646

[59] Ha YJ Shin YJ Tak KH Park JL Kim JH Lee JL . Reduced expression of alanyl aminopeptidase is a robust biomarker of non-familial adenomatous polyposis and non-hereditary nonpolyposis colorectal cancer syndrome early-onset colorectal cancer. Cancer Med. (2023) 12:10091–104. doi: 10.1002/cam4.5675 PMC10166950 36748835

[60] Yang J Zhao Y Yuan R Wang Y Wang S Chang Z . Identifying individualized prognostic signature and unraveling the molecular mechanism of recurrence in early-onset colorectal cancer. Eur J Med Res. (2023) 28:533. doi: 10.1186/s40001-023-01491-y 37986009 PMC10658991

[61] Furuhashi S Bustos MA Mizuno S Ryu S Naeini Y Bilchik AJ . Spatial profiling of cancer-associated fibroblasts of sporadic early onset colon cancer microenvironment. NPJ Precis Oncol. (2023) 7:118. doi: 10.1038/s41698-023-00474-w 37964075 PMC10645739

[62] Chang SH Patel N Du M Liang PS . Trends in early-onset vs late-onset colorectal cancer incidence by race/ethnicity in the United States cancer statistics database. Clin Gastroenterol Hepatol. (2022) 20:e1365–77. doi: 10.1016/j.cgh.2021.07.035 PMC8789949 34325062

[63] Wang R Wang MJ Ping J . Clinicopathological features and survival outcomes of colorectal cancer in young versus elderly: A population-based cohort study of SEER 9 registries data (1988-2011). Med (Baltimore). (2015) 94:e1402. doi: 10.1097/MD.0000000000001402 PMC4616510 26334895

[64] McClelland PHT Liu T Ozuner G . Early-onset colorectal cancer in patients under 50 years of age: demographics, disease characteristics, and survival. Clin Colorectal Cancer. (2022) 21:e135–44. doi: 10.1016/j.clcc.2021.11.003 34972664

[65] Saraiva MR Rosa I Claro I . Early-onset colorectal cancer: A review of current knowledge. World J Gastroenterol. (2023) 29:1289–303. doi: 10.3748/wjg.v29.i8.1289 PMC10011966 36925459

[66] Cavestro GM Mannucci A Balaguer F Hampel H Kupfer SS Repici A . Delphi initiative for early-onset colorectal cancer (DIRECt) international management guidelines. Clin Gastroenterol Hepatol. (2023) 21:581–603. doi: 10.1016/j.cgh.2022.12.006 36549470 PMC11207185

[67] Kneuertz PJ Chang GJ Hu CY Rodriguez-Bigas MA Eng C Vilar E . Overtreatment of young adults with colon cancer: more intense treatments with unmatched survival gains. JAMA Surg. (2015) 150:402–9. doi: 10.1001/jamasurg.2014.3572 25806815

[68] Abdelsattar ZM Wong SL Regenbogen SE Jomaa DM Hardiman KM Hendren S . Colorectal cancer outcomes and treatment patterns in patients too young for average-risk screening. Cancer. (2016) 122:929–34. doi: 10.1002/cncr.29716 PMC4777631 26808454

[69] Kanter K Fish M Mauri G Horick NK Allen JN Blaszkowsky LS . Care patterns and overall survival in patients with early-onset metastatic colorectal cancer. JCO Oncol Pract. (2021) 17:e1846–55. doi: 10.1200/OP.20.01010 34043449

[70] NCCN . Guidelines Detail (2023). Available online at: https://www.nccn.org/guidelines/guidelines-detail.

[71] Afrăsânie VA Marinca MV Alexa-Stratulat T Gafton B Păduraru M Adavidoaiei AM . KRAS, NRAS, BRAF, HER2 and microsatellite instability in metastatic colorectal cancer - practical implications for the clinician. Radiol Oncol. (2019) 53:265–74. doi: 10.2478/raon-2019-0033 PMC6765160 31553708

[72] Taieb J Zaanan A Le Malicot K Julié C Blons H Mineur L . Prognostic effect of BRAF and KRAS mutations in patients with stage III colon cancer treated with leucovorin, fluorouracil, and oxaliplatin with or without cetuximab: A *post hoc* analysis of the PETACC-8 trial. JAMA Oncol. (2016) 2:643–53. doi: 10.1001/jamaoncol.2015.5225 26768652

[73] Jácome AA Vreeland TJ Johnson B Kawaguchi Y Wei SH Nancy You Y . The prognostic impact of RAS on overall survival following liver resection in early versus late-onset colorectal cancer patients. Br J Cancer. (2021) 124:797–804. doi: 10.1038/s41416-020-01169-w 33208919 PMC7884678

[74] Aljehani MA Bien J Lee JSH Fisher GA Lin AY . KRAS sequence variation as prognostic marker in patients with young- vs late-onset colorectal cancer. JAMA Netw Open. (2023) 6:e2345801. doi: 10.1001/jamanetworkopen.2023.45801 38032636 PMC10690478

[75] Khan SA Morris M Idrees K Gimbel MI Rosenberg S Zeng Z . Colorectal cancer in the very young: a comparative study of tumor markers, pathology and survival in early onset and adult onset patients. J Pediatr Surg. (2016) 51:1812–7. doi: 10.1016/j.jpedsurg.2016.07.015 PMC5312708 27558481

[76] Irahara N Baba Y Nosho K Shima K Yan L Dias-Santagata D . NRAS mutations are rare in colorectal cancer. Diagn Mol Pathol. (2010) 19:157–63. doi: 10.1097/PDM.0b013e3181c93fd1 PMC2929976 20736745

[77] Capper D Voigt A Bozukova G Ahadova A Kickingereder P von Deimling A . BRAF V600E-specific immunohistochemistry for the exclusion of Lynch syndrome in MSI-H colorectal cancer. Int J Cancer. (2013) 133:1624–30. doi: 10.1002/ijc.28183 23553055

[78] Danielsen SA Eide PW Nesbakken A Guren T Leithe E Lothe RA . Portrait of the PI3K/AKT pathway in colorectal cancer. Biochim Biophys Acta. (2015) 1855:104–21. doi: 10.1016/j.bbcan.2014.09.008 25450577

[79] Li QH Wang YZ Tu J Liu CW Yuan YJ Lin R . Anti-EGFR therapy in metastatic colorectal cancer: mechanisms and potential regimens of drug resistance. Gastroenterol Rep (Oxf). (2020) 8:179–91. doi: 10.1093/gastro/goaa026 PMC7333932 32665850

[80] Nakayama M Oshima M . Mutant p53 in colon cancer. J Mol Cell Biol. (2019) 11:267–76. doi: 10.1093/jmcb/mjy075 PMC6487790 30496442

[81] Michel M Kaps L Maderer A Galle PR Moehler M . The role of p53 dysfunction in colorectal cancer and its implication for therapy. Cancers (Basel). (2021) 13:2296. doi: 10.3390/cancers13102296 34064974 PMC8150459

[82] Aghabozorgi AS Bahreyni A Soleimani A Bahrami A Khazaei M Ferns GA . Role of adenomatous polyposis coli (APC) gene mutations in the pathogenesis of colorectal cancer; current status and perspectives. Biochimie. (2019) 157:64–71. doi: 10.1016/j.biochi.2018.11.003 30414835

[83] Li J Ma X Chakravarti D Shalapour S DePinho RA . Genetic and biological hallmarks of colorectal cancer. Genes Dev. (2021) 35:787–820. doi: 10.1101/gad.348226.120 34074695 PMC8168558

[84] Sinicrope FA . Lynch syndrome-associated colorectal cancer. N Engl J Med. (2018) 379:764–73. doi: 10.1056/NEJMcp1714533 30134129

[85] McRonald FE Pethick J Santaniello F Shand B Tyson A Tulloch O . Identification of people with Lynch syndrome from those presenting with colorectal cancer in England: baseline analysis of the diagnostic pathway. Eur J Hum Genet. (2024). doi: 10.1038/s41431-024-01550-w PMC11061113 38355963

[86] Mezzapesa M Losurdo G Celiberto F Rizzi S d’Amati A Piscitelli D . Serrated colorectal lesions: an up-to-date review from histological pattern to molecular pathogenesis. Int J Mol Sci. (2022) 23:4461. doi: 10.3390/ijms23084461 35457279 PMC9032676

[87] Barresi V Reggiani Bonetti L Ieni A Caruso RA Tuccari G . Histological grading in colorectal cancer: new insights and perspectives. Histol Histopathol. (2015) 30:1059–67. doi: 10.14670/HH-11-633 26004398

[88] Fadel MG Malietzis G Constantinides V Pellino G Tekkis P Kontovounisios C . Clinicopathological factors and survival outcomes of signet-ring cell and mucinous carcinoma versus adenocarcinoma of the colon and rectum: a systematic review and meta-analysis. Discovery Oncol. (2021) 12:5. doi: 10.1007/s12672-021-00398-6 PMC8762524 35201441

[89] Luo C Cen S Ding G Wu W . Mucinous colorectal adenocarcinoma: clinical pathology and treatment options. Cancer Commun (Lond). (2019) 39:13. doi: 10.1186/s40880-019-0361-0 30922401 PMC6440160

[90] Gabriel E Attwood K Al-Sukhni E Erwin D Boland P Nurkin S . Age-related rates of colorectal cancer and the factors associated with overall survival. J Gastrointest Oncol. (2018) 9:96–110. doi: 10.21037/jgo 29564176 PMC5848036

[91] Ding X Yang X Wu D Huang Y Dai Y Li J . Nomogram predicting the cancer-specific survival of early-onset colorectal cancer patients with synchronous liver metastasis: a population-based study. Int J Colorectal Dis. (2022) 37:1309–19. doi: 10.1007/s00384-022-04175-x 35524790

[92] Chen Y He L Lu X Tang Y Luo G Chen Y . Causes of death among early-onset colorectal cancer population in the United States: a large population-based study. Front Oncol. (2023) 13:1094493. doi: 10.3389/fonc.2023.1094493 37168371 PMC10166590

[93] Benesch MGK Mathieson A O’Brien SBL . Effects of tumor localization, age, and stage on the outcomes of gastric and colorectal signet ring cell adenocarcinomas. Cancers (Basel). (2023) 15:714. doi: 10.3390/cancers15030714 36765680 PMC9913295

[94] De Renzi G Gaballo G Gazzaniga P Nicolazzo C . Molecular biomarkers according to primary tumor location in colorectal cancer: current standard and new insights. Oncology. (2021) 99:135–43. doi: 10.1159/000510944 33130682

[95] Cheng E Blackburn HN Ng K Spiegelman D Irwin ML Ma X . Analysis of survival among adults with early-onset colorectal cancer in the national cancer database. JAMA Netw Open. (2021) 4:e2112539. doi: 10.1001/jamanetworkopen.2021.12539 34132794 PMC8209612

[96] Charlton ME Kahl AR Greenbaum AA Karlitz JJ Lin C Lynch CF . KRAS testing, tumor location, and survival in patients with stage IV colorectal cancer: SEER 2010-2013. J Natl Compr Canc Netw. (2017) 15:1484–93. doi: 10.6004/jnccn.2017.7011 PMC7458121 29223986

[97] Xie MZ Li JL Cai ZM Li KZ Hu BL . Impact of primary colorectal Cancer location on the KRAS status and its prognostic value. BMC Gastroenterol. (2019) 19:46. doi: 10.1186/s12876-019-0965-5 30917791 PMC6437985

[98] Lee MS Menter DG Kopetz S . Right versus left colon cancer biology: integrating the consensus molecular subtypes. J Natl Compr Canc Netw. (2017) 15:411–9. doi: 10.6004/jnccn.2017.0038 28275039

[99] Foppa C Tamburello S Maroli A Carvello M Poliani L Laghi L . Early age of onset is an independent predictor for worse disease-free survival in sporadic rectal cancer patients. A comparative analysis of 980 consecutive patients. Eur J Surg Oncol. (2022) 48:857–63. doi: 10.1016/j.ejso.2021.10.021 34740480

[100] Laskar RS Ghosh SK Talukdar FR . Rectal cancer profiling identifies distinct subtypes in India based on age at onset, genetic, epigenetic and clinicopathological characteristics. Mol Carcinog. (2015) 54:1786–95. doi: 10.1002/mc.22250 25418895

[101] Perea J García JL Corchete L Tapial S Olmedillas-López S Vivas A . A clinico-pathological and molecular analysis reveals differences between solitary (early and late-onset) and synchronous rectal cancer. Sci Rep. (2021) 11:2202. doi: 10.1038/s41598-020-79118-z 33500439 PMC7838158

[102] Spolverato G Fassan M Scarpa M Stepanyan A De Simoni O Scognamiglio F . IMMUNOREACT 6: weak immune surveillance characterizes early-onset rectal cancer. Br J Surg. (2023) 110:1490–501. doi: 10.1093/bjs/znad219 37478362

[103] Ugai T Haruki K Harrison TA Cao Y Qu C Chan AT . Molecular characteristics of early-onset colorectal cancer according to detailed anatomical locations: comparison with later-onset cases. Am J Gastroenterol. (2023) 118:712–26. doi: 10.14309/ajg.0000000000002171 PMC10065351 36707929

[104] Baran B Mert Ozupek N Yerli Tetik N Acar E Bekcioglu O Baskin Y . Difference between left-sided and right-sided colorectal cancer: A focused review of literature. Gastroenterol Res. (2018) 11:264–73. doi: 10.14740/gr1062w PMC6089587 30116425

[105] Galon J Costes A Sanchez-Cabo F Kirilovsky A Mlecnik B Lagorce-Pagès C . Type, density, and location of immune cells within human colorectal tumors predict clinical outcome. Science. (2006) 313:1960–4. doi: 10.1126/science.1129139 17008531

[106] Angell HK Bruni D Barrett JC Herbst R Galon J . The immunoscore: colon cancer and beyond. Clin Cancer Res. (2020) 26:332–9. doi: 10.1158/1078-0432.CCR-18-1851 31413009

[107] Ten Hoorn S de Back TR Sommeijer DW Vermeulen L . Clinical value of consensus molecular subtypes in colorectal cancer: A systematic review and meta-analysis. J Natl Cancer Inst. (2022) 114:503–16. doi: 10.1093/jnci/djab106 PMC9002278 34077519

