# Supplementary material for: The histological and molecular characteristics of early-onset colorectal cancer: a systematic review and meta-analysis
Source: Front Oncol. 2024 Apr 26;14:1349572. doi: 10.3389/fonc.2024.1349572 (PMC11082351; doi:10.3389/fonc.2024.1349572)
Supplement: Supplementary file 1 [file DataSheet_1.docx]

**Supplementary Figure S1: Odds ratios for *PIK3CA* mutation in early-onset colorectal cancer**

**

**

Data presented as odds ratios (95% confidence interval) for *PIK3CA* mutation in early-onset relative to late-onset colorectal cancer. The pooled odds ratio is obtained via a random effects model using inverse variance weighting.

Abbreviations: AACR – American Association for Cancer Research; CI – confidence interval; EO-CRC – early-onset colorectal cancer; MDACC – MD Anderson Cancer Center; MSI – microsatellite instability; MSS – microsatellite stable

**Supplementary Figure S2: Odds ratios for *HER2* amplifications in early-onset colorectal cancer**

**

**

Data presented as odds ratios (95% confidence interval) for *HER2* amplifications in early-onset relative to late-onset colorectal cancer. The pooled odds ratio is obtained via a random effects model using inverse variance weighting.

Abbreviations: CI – confidence interval; EO-CRC – early-onset colorectal cancer;

**Supplementary Figure S3: Odds ratios for the CpG island methylator phenotype in early-onset colorectal cancer**

**

**

Data presented as odds ratios (95% confidence interval) for the CpG island methylator phenotype in early-onset relative to late-onset colorectal cancer. The pooled odds ratio is obtained via a random effects model using inverse variance weighting.

Abbreviations: AACR – American Association for Cancer Research; CI – confidence interval; EO-CRC – early-onset colorectal cancer; MDACC – MD Anderson Cancer Center; MSI – microsatellite instability; MSS – microsatellite stable

**Supplementary Figure S4: Odds ratios for microsatellite instability in early-onset colorectal cancer**

**

**

Data presented as odds ratios (95% confidence interval) for microsatellite instability in early-onset relative to late-onset colorectal cancer. The pooled odds ratio is obtained via a random effects model using inverse variance weighting.

Abbreviations: CI – confidence interval; EO-CRC – early-onset colorectal cancer; PCRC – primary unifocal colorectal cancer; SCRC – synchronous colorectal cancer

**Supplementary Figure S5: Odds ratios for high tumor grade or poorly differentiated tumors in early-onset colorectal cancer**

**

**

Data presented as odds ratios (95% confidence interval) for high grade or poorly differentiated tumors in early-onset relative to late-onset colorectal cancer. The pooled odds ratio is obtained via a random effects model using inverse variance weighting.

Abbreviations: CI – confidence interval; EO-CRC – early-onset colorectal cancer; LC – Linkoping Cancer database; MSI – microsatellite instability; MSS – microsatellite stable; PCRC – primary unifocal colorectal cancer; SCRC – synchronous colorectal cancer; SEER – Surveillance, Epidemiology, and End Results Program; SYSU – Sun Yat-sen University; WC – West China

**Supplementary Figure S6: Odds ratios for tumors with mucinous histology in early-onset colorectal cancer**

**

**

Data presented as odds ratios (95% confidence interval) for tumors with mucinous histology in early-onset relative to late-onset colorectal cancer. The pooled odds ratio is obtained via a random effects model using inverse variance weighting.

Abbreviations: CI – confidence interval; EO-CRC – early onset colorectal cancer; MSI – microsatellite instability; MSS – microsatellite stable; PCRC – primary unifocal colorectal cancer; SCRC – synchronous colorectal cancer; SEER – Surveillance, Epidemiology, and End Results Program, WC – West China

**Supplementary Figure S7: Odds ratios for tumors with signet ring histology in early-onset colorectal cancer**





Data presented as odds ratios (95% confidence interval) for tumors with signet ring histology in early-onset relative to late-onset colorectal cancer. The pooled odds ratio is obtained via a random effects model using inverse variance weighting.

Abbreviations: CI – confidence interval; EO-CRC – early-onset colorectal cancer; PCRC – primary unifocal colorectal cancer; SCRC – synchronous colorectal cancer
