# Supplementary material for: The histological and molecular characteristics of early-onset colorectal cancer: a systematic review and meta-analysis
Source: Front Oncol. 2024 Apr 26;14:1349572. doi: 10.3389/fonc.2024.1349572 (PMC11082351; doi:10.3389/fonc.2024.1349572)
Supplement: Supplementary file 2 [file Table_1.docx]

**Article title:** The clinicopathological and molecular characteristics of early-onset colorectal cancer: a systematic review

**Authors:** Thomas Lawler^1^, Lisa Parlato^2^, Shaneda Warren Andersen^1,2^

**Affiliations:**

^1^Carbone Cancer Center, University of Wisconsin-Madison, Madison, WI, USA

^2^School of Medicine and Public Health, Department of Population Health Sciences, University of Wisconsin-Madison, Madison, WI, USA

**Corresponding author:**

Shaneda Warren Andersen, PhD

Address: Suite 1007B, WARF, 610 Walnut Street, Madison, WI, 53726

Email: snandersen@wisc.edu

Phone number: 608-265-8257

**Supplementary Table S1: Pubmed literature search query**

(“colorectal neoplasms”[mesh] OR “colorectal cancer” OR “colonic neoplasms”[mesh] OR “colon cancer” OR “rectal neoplasms”[mesh] or “rectal cancer”) AND (“age of onset”[mesh] OR “early-onset”[tiab] OR “early onset”[tiab] OR “young-onset”[tiab] OR “young onset”[tiab] OR “50 years”[tiab] or “age 50”[tiab]) AND ((biomark* OR mark* OR character*) OR (“neoplasm staging”[mesh] OR “neoplasm grading”[mesh] OR TNM[tiab] OR histo*[tiab] OR metasta*[tiab] OR differentiat* OR budding[tiab] OR heterogeneity[tiab] OR IITH[tiab] OR IPTH[tiab] OR micropapillary[tiab] OR cribriform[tiab] OR mucin*[tiab] or MUC1[tiab] or MUC2[tiab] or MUC-1[tiab] OR MUC-2[tiab] OR signet*[tiab] OR pedunculat*[tiab] OR serrated[tiab] OR sessile[tiab] OR hyperplastic[tiab] OR dysplas*[tiab] OR “microvessel density”[tiab] OR MVD[tiab] or “angiolymphatic invasion”[tiab] or “lymphatic invasion”[tiab] or LVI[tiab] or “perineural invasion”[tiab] or PNI[tiab] or infiltrat*[tiab] OR non-polypoid[tiab]) OR (“molecular subtype”[tiab] OR “molecular subtypes”[tiab] OR “consensus molecular subtype”[tiab] OR “consensus molecular subtypes”[tiab] OR CRCSC[tiab] OR CMS[tiab] OR CMS1[tiab] OR CMS2[tiab] OR CMS3[tiab] OR CMS4[tiab] OR MSI[tiab] OR MSI-H[tiab] OR “microsatellite instability”[tiab] OR CIN[tiab] OR “chromosomal instability”[tiab] OR CIMP[tiab] OR CIMP-H[tiab] OR “loss of heterozygosity”[tiab] OR “LOH”[tiab] OR “allelic loss”[tiab] OR “allele loss”[tiab] OR 18q* OR “CpG Island methylator phenotype”[tiab] OR heterogeneity[tiab]) OR (“mutation”[tiab] or “somatic mutation”[tiab] or heterogeneity[tiab] or mesenchymal[tiab] or EMT[tiab] or MACS[tiab] or APC or IMP3 or S100A2 or TNIK or NTRK or ALK or kinase[tiab] or MCC or DCC or SMAD* or CABLES* or TGFB*[tiab] or TGFβ[tiab] or POLE* or EGFR*[tiab] or BRAF* or B-RAF* or RAF or RAS or KRAS or K-RAS or NRAS or N-RAS or TP53 or P53 or PVT1 or PIK3CA or PTEN or ACVR2* or VEGF*[tiab] or EPHA6 or FBXW7 or CHD5 or PDCD4 or IGF[tiab] or MAPK[tiab] or CTNNB* or DPYD or DPD or SCNA or CNA or SNV or SCNV or MACC*) OR (epigenetic*[tiab] or upregulat* [tiab] or up-regulat*[tiab] or downregulat* [tiab] or down-regulat*[tiab] or regulat*[tiab] or RNA[tiab] or lncRNA*[tiab] or ncRNA*[tiab] or microRNA*[tiab] or miRNA*[tiab] or histone or methylat*[tiab] or hypomethylat*[tiab] or hypermethylat*[tiab] or express*[tiab] or CDX2 or CEA[tiab] or SAA[tiab] or PCNA[tiab] or HPP1[tiab] or HTLF[tiab] or cfDNA[tiab] or cf-DNA[tiab] or ctDNA[tiab] or ct-DNA[tiab] or CTC[tiab] or NDRG* or MLH1 or SA1 or ERBB* or HER2 or HER-2 or MET or P27 or P27kip1 or Ki67 or BCL2 or BCL-2 or SLC5A8 or STAT3 or Ezrin or COX-2 or ERCC* or “thymidylate synthase”or TS or “stem cell” or “stem cells” or CSC or CSCs or hypoxia-inducible* or hypoxia* or ADCC or hTERT or TET or epiregulin or amphiregulin or CXCR* or CXCL* or Claudin* or COMP or MGMT or LINE-1) OR (“immunity”[mesh] OR immun*[tiab] OR “B-Lymphocytes”[mesh] OR Bcell OR B-cell OR “T-Lymphocytes”[mesh] OR Tcell OR T-cell OR “Natural Killer T-cells”[mesh] OR “Natural Killer Cells” OR “NK cells” OR “Dendritic cells”[mesh] OR “Dendritic cells” OR “Macrophages”[mesh] OR macrophages OR “Myeloid cells”[mesh] OR “Myeloid cells” OR “T-lymphocytes, regulatory”[mesh] OR “Regulatory T cells” OR “Treg” OR “T-reg” OR “immunoscore”))
