# Supplementary material for: The histological and molecular characteristics of early-onset colorectal cancer: a systematic review and meta-analysis
Source: Front Oncol. 2024 Apr 26;14:1349572. doi: 10.3389/fonc.2024.1349572 (PMC11082351; doi:10.3389/fonc.2024.1349572)
Supplement: Supplementary file 4 [file Table_3.docx]

**Article title:** The clinicopathological and molecular characteristics of early-onset colorectal cancer: a systematic review

**Authors:** Thomas Lawler^1^, Lisa Parlato^2^, Shaneda Warren Andersen^1,2^

**Affiliations:**

^1^Carbone Cancer Center, University of Wisconsin-Madison, Madison, WI, USA

^2^School of Medicine and Public Health, Department of Population Health Sciences, University of Wisconsin-Madison, Madison, WI, USA

**Corresponding author:**

Shaneda Warren Andersen, PhD

Address: Suite 1007B, WARF, 610 Walnut Street, Madison, WI, 53726

Email: snandersen@wisc.edu

Phone number: 608-265-8257

| **Supplementary Table S3: Results of leave-one-out sensitivity analysis for each marker included in the meta-analysis** | | | | |
| --- | --- | --- | --- | --- |
|  | HR (range) | Lower CI (range) | Upper CI (range) | P-value (range) |
| *KRAS* mutation | 0.89-0.92 | 0.84-0.86 | 0.94-0.99 | P < .001 - .02 |
| *BRAF* mutation | 0.60-0.70 | 0.49-0.58 | 0.75-0.84 | P < .001 for all |
| *NRAS* mutation | 0.87-0.90 | 0.76-0.78 | 0.99-1.04 | P = .03 - .16 |
| *APC* mutation | 0.67-0.73 | 0.55-0.63 | 0.81-0.88 | P < .001 for all |
| *TP53* mutation | 1.32-1.38 | 1.20-1.30 | 1.43-1.48 | P < .001 for all |
| *PTEN* mutation | 1.30-2.01 | 0.89-1.18 | 1.88-3.25 | P = .009 - .18 |
| *PIK3CA* mutation | 0.92-0.97 | 0.84-0.87 | 1.00-1.09 | P > .05 for all |
| *HER2* amplification | 1.22-1.88 | 0.64-0.97 | 2.19 -4.50 | P > .05 for all |
| CIMP mutation | 0.19-0.41 | 0.07-0.23 | 0.49-0.75 | P < .001 - .004 |
| MSI | 1.29-1.38 | 1.09-1.16 | 1.53-1.63 | P < .01 for all |
| High tumor grade | 1.19-1.23 | 1.14-1.17 | 1.23-1.29 | P < .001 for all |
| Mucinous histology | 1.20-1.24 | 1.15-1.17 | 1.26-1.31 | P < .001 for all |
| Signet ring histology | 2.23-2.37 | 2.01-2.14 | 2.48-2.68 | P < .001 for all |
| Abbreviations: CIMP – CpG Island Methylator Phenotype; MSI – microsatellite instability | | | | |
